# A Wearable Soft Fabric Sleeve for Upper Limb Augmentation †

**DOI:** 10.3390/s21227638

**Published:** 2021-11-17

**Authors:** Trung Thien Hoang, Luke Sy, Mattia Bussu, Mai Thanh Thai, Harrison Low, Phuoc Thien Phan, James Davies, Chi Cong Nguyen, Nigel H. Lovell, Thanh Nho Do

**Affiliations:** 1 Graduate School of Biomedical Engineering, Faculty of Engineering, University of New South Wales, Sydney 2052, Australia; trungthien.hoang@unsw.edu.au (T.T.H.); l.sy@unsw.edu.au (L.S.); maithanh.thai@unsw.edu.au (M.T.T.); h.low@unsw.edu.au (H.L.); phuoc_thien.phan@unsw.edu.au (P.T.P.); j.j.davies@student.unsw.edu.au (J.D.); cong.c.nguyen@unsw.edu.au (C.C.N.); n.lovell@unsw.edu.au (N.H.L.); 2 Institute of Robotics and Intelligent Systems, Eidgenössische Technische Hochschule Zürich, 8092 Zurich, Switzerland; mbussu@student.ethz.ch

**Keywords:** soft robotics, wearable devices, upper limb augmentation, soft sensors, soft actuators, hysteresis modelling, fabric sleeve, liquid metal

## Abstract

Soft actuators (SAs) have been used in many compliant robotic structure and wearable devices, due to their safe interaction with the wearers. Despite advances, the capability of current SAs is limited by scalability, high hysteresis, and slow responses. In this paper, a new class of soft, scalable, and high-aspect ratio fiber-reinforced hydraulic SAs is introduced. The new SA uses a simple fabrication process of insertion where a hollow elastic rubber tube is directly inserted into a constrained hollow coil, eliminating the need for the manual wrapping of an inextensible fiber around a long elastic structure. To provide high adaptation to the user skin for wearable applications, the new SAs are integrated into flexible fabrics to form a wearable fabric sleeve. To monitor the SA elongation, a soft liquid metal-based fabric piezoresistive sensor is also developed. To capture the nonlinear hysteresis of the SA, a novel asymmetric hysteresis model which only requires five model parameters in its structure is developed and experimentally validated. The new SAs-driven wearable robotic sleeve is scalable, highly flexible, and lightweight. It can also produce a large amount of force of around 23 N per muscle at around 30% elongation, to provide useful assistance to the human upper limbs. Experimental results show that the soft fabric sleeve can augment a user’s performance when working against a load, evidenced by a significant reduction on the muscular effort, as monitored by electromyogram (EMG) signals. The performance of the developed SAs, soft fabric sleeve, soft liquid metal fabric sensor, and nonlinear hysteresis model reveal that they can effectively modulate the level of assistance for the wearer. The new technologies obtained from this work can be potentially implemented in emerging assistive applications, such as rehabilitation, defense, and industry.

## 1. Introduction

Over the past two decades, an increasing number of academic and industry groups have dedicated themselves to their work on the development of upper limb exoskeletons for rehabilitation and power augmentation applications [1,2]. Physically weak individuals due to aged, injured, or handicapped situations have been identified as the primary beneficiaries of this technology. The growth of exoskeleton technology has also been prompted by an endeavor of the current healthcare system to benefit stroke victims, who often experience the chronic impairment of upper limbs, with an optimum rehabilitation therapy [3,4]. The individuals, who suffer from partial or full loss of motor function, tend to lose their ability to perform basic activities of daily living (ADL), which causes their independence to be restricted and their quality of life to be significantly degraded. Rehabilitation intervention can be helpful in these cases when it comes to helping them regain various motor skills [1,2,5,6]. In fact, it has been proven that upper limb exercises that are highly intensive and task-specific, such as active and repetitive movements, are the most effective methods of therapy for stroke victims to restore their arm function [7]. Assistive robots are able to provide this kind of training, allowing patients to semi-autonomously practice their movements, thus facilitating the therapist’s job [8,9]. Several clinical studies revealed the positive effects of robot-assisted rehabilitation, which are found to be at least as effective as conventional intensive therapy [10,11,12]. Although rigid exoskeleton systems are beneficial for applications requiring high force/torque transmission, or where portability is not a necessity, such as in the case of on-site rehabilitation [13], their large size, high weight and rigidity make this design archetype suboptimal for everyday use. In addition, the lack of compliance with the wearer’s body poses safety concerns and can cause discomfort during the human–machine interaction. The practical challenges faced in translating lab-based rigid-bodied systems for real-world applications have driven the research to a new design philosophy that sees the replacement of external rigid frames with soft, clothing-like frames. The resulting soft-bodied exoskeletons display some significant advantages, such as reduced weight, low profile, and increased comfort. These are key requirements for assistive wearable robots that are designed to facilitate the functional movement of the wearer during ADLs [14,15,16].

Nowadays, the most conventional approach to power soft exoskeletons is based on tendon-driven actuators, which normally consist of an electrical motor and Bowden cables [17,18,19]. Despite providing a lightweight and flexible solution, cable transmission systems introduce major disadvantages, such as the presence of non-linearities of friction and backlash hysteresis that affect their control accuracy and reduce the energy efficiency [20,21,22,23]. In recent years, the upswing in soft robotics has permeated into the field of assistive wearable devices with the introduction of new soft actuation technologies [13,24,25,26,27,28,29,30,31,32,33]. Their inherent compliance, providing an opportunity to overcome the safety concerns and ergonomic limitations that are typical of rigid-bodied robots. The most common soft robotic actuators are flexible fluidic actuators (FFAs) [34,35,36,37,38,39,40,41,42,43], shape memory materials (SMMs), and electroactive polymers (EAPs) [26,27,32,44]. Most FFAs are driven by air pressure which is known as a pneumatic artificial muscle (PAM). Hydraulic actuation is used when high force capability, low noise, and low nonlinearity are required [32]. The most established and commonly used PAMs are McKibben actuators, which consist of an airtight internal bladder surrounded by a braided mesh shell made from flexible yet inextensible threads. When the rubber tube is pressurised, the woven nature of the sheath results in axial shortening and radial expansion of the actuator, converting input pressure into mechanical work [45,46]. Other variations of PAMs have been developed, such as pleated PAMs (PPAM) [28], vacuum-powered PAMs [47,48], and inverse PAMs (IPAM) [25,49].

Major attractions of PAMs are the high-power to weight ratio and the direct connection to the structure that they power, thus excluding the need for gearing, which is associated with unwanted phenomena in the system, such as backlash and extra inertia [50,51]. However, there is a challenge to develop a fluid-driven artificial muscle that is lightweight, high strain, and is long in length, with low hysteresis with physical structure, similar to conventional cable mechanisms. For other types of actuations, the SMMs comprising of alloys (SMAs), polymers (SMPs) and composites are capable of recovering a memorized shape after a plastic deformation under external stimuli. The SMAs can produce high force with specific power similar to human muscle [44]. The major limitations of current SMM-based technologies are low efficiency, low strain, and low speed. EAPs are another smart material of relevance to exoskeletons [44] which are a type of active polymers that are able to undergo shape deformation when electric fields are applied [44]. The most popular types of EAPs are dielectric elastomer actuators (DEAs) and ionic polymer-metal composites (IPMCs) actuators. However, manufacturing a big enough actuator with high strain to produce a useful amount of power and force is challenging.

Nonlinear hysteresis in existing soft artificial muscles (SAMs) also poses many challenges to precisely control the system for optimal outcomes. The development of effective hysteresis models to capture the nonlinearity of the SAMs has been an active research area in recent decades, ranging from wearable assistive devices to surgical robots [22,24,51,52]. Many studies have attempted to develop effective hysteresis models using discrete approaches such as Preisach, Maxwell–Slip, and Prandtl–Ishlinskii (P-I) models. However, their accuracy closely depends on the selection of a number of hysteresis elements, which requires a complex identification process, and significant computational time [50,51]. In contrast to the discrete approaches, continuous models, such as the Bouc–Wen model or its variants, are potential candidates to describe the dynamic hysteresis of nonlinear systems, with less complexity and ease of control implementation [53]. These models capture the hysteresis curves by using continuous state variables and shape parameters of the hysteresis loops. Despite advances, there is a trade-off between the number of model variables and their accuracy [50,51].

Though still at an early stage, the successful integration of soft actuation and sensing technologies in wearable assistive devices is seeing increasing endorsement. However, major limitations, such as low strain or low force, have hampered their widespread adoption, leaving room for further improvement. Previously, we have developed a simple version of the soft robotic sleeve that could provide useful support to the wearer [54]. However, we have not completely described full characterizations for the soft muscles, such as the effect of outer constrained coil on the hysteresis profile, the nonlinear hysteresis model with a small number of model parameters, the degree of hysteresis, and the use of soft fabric sensors for monitoring muscle strain or elongation. In this paper, we re-introduce a new class of soft artificial actuators that are lightweight, highly compliant, scalable, and with a high aspect ratio (length/diameter > 300). Unlike traditional McKibben actuators, the flexibility and scalability of the developed soft artificial actuators introduced in this paper allow for the organization into larger structures, such as the sheet-like configurations of braiding or weaving yarn used in conventional apparel engineering. In this way, highly distributed forces can be produced, while maintaining high conformability to complex skin surfaces via a flexible fabric interface. These features make this new actuator exceptionally suitable for use in wearable devices that can be worn as human clothing. In addition, we also developed a new liquid metal-based soft fabric skin sensor that can precisely detect the muscle strain for feedback control purposes, or for monitoring gait posture. 

To predict the hysteresis nonlinearity between the input driving source and the soft actuator (SA) elongation, we also designed and experimentally validated a new asymmetric hysteresis model, which has fewer model parameters in its structure compared to a conventional Bouc–Wen model [53], while offering great accuracy to capture the complex hysteresis loop. To this end, we aimed to realize a wearable upper limb fabric suit with an integrated soft sensor that assisted elbow flexion and extension movements, as well as maintaining posture with no additional metallic energy. The developed wearable robotic sleeve could be potentially used to support the user in activities of daily living (ADLs) or augment lifting and carrying ability in the workplace. The further development of such an exoskeleton could also find applications in rehabilitation by extending the therapy of hemiparetic upper limbs outside the clinical setting and into the patient’s home, with benefits to the cost and accessibility of care.

## 2. Materials and Methods

### 2.1. Overview of the Soft Actuator and Soft Fabric Sleeve

**Figure 1:**
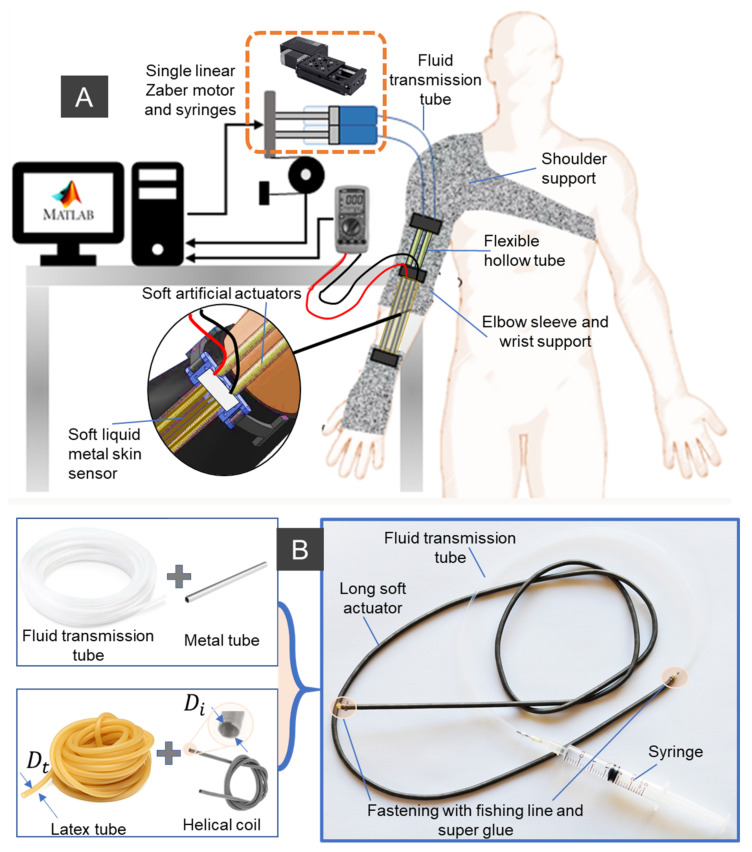
Soft wearable fabric sleeve for upper limb assistance. (**A**) Conceptual design with integrated components, including soft liquid metal-based skin sensor, soft artificial actuators and their guide tube, elbow support, wrist support, and shoulder support. It is noted that each muscle is connected to a miniature syringe, and the array of these muscles is driven by a single Zaber DC motor. (**B**) Fabrication process for the high aspect ratio soft actuator and its prototype (length/outer diameter = 1000 mm/3.15 mm ~317).

The new robotic fabric sleeve consists of a fabric shoulder, a fabric elbow sleeve, a fabric wrist support, miniature SAs, a soft liquid metal (LM)-based skin fabric sensor, and an actuation stage (DC motors, miniature syringes, and linear ball screws). Details are shown in Figure 1. The soft fabric sleeve is devoted to transferring the axial tension force from the SAs to torque applied to the limb joint, and thus assist with upper limb motion. Within the soft fabric sleeve, there is an array of SAs, which are arranged in parallel and connected to miniature syringes via fluid transmission tubes. According to different required loads, there will be a double or an array of miniature SAs that can be woven to form a sheet-like soft artificial actuator. To drive the SAs, a linear DC motor which is externally located will control the axial displacement of a miniature hydraulic syringe. The hydraulic element can be water or hydraulic oil. To monitor the actuator strain for bending motion feedback, a soft piezoresistive sensor made from a soft silicone microtube and LM eutectic gallium indium (EGaIn), which is sandwiched in between two stretchable fabric layers, was used. For the fabric sleeve design, the SAs are held together and lined up using 3D-printed locks, which function as anchor points. The upper lock and middle lock are positioned on the proximal side of the elbow joint, while the lower lock sits on the distal side. To achieve high elongation or contraction force, each SA can be routed inside a flexible yet inextensible tube acting as a guided sheath where the SA slides freely during the operation. The flexible guided sheaths are fixed onto the upper limb, and therefore their shape configuration is unchanged during the operation. The SA can extend or contract under the applied hydraulic pressure, where the contraction force is higher than the extension force. Detailed operating principles of the SA will be given in the next section. In our design, the resulting tensile force generated by the SA under a reduced fluid pressure pulls the distal anchor point closer to the arm, where it generates a bending moment around the elbow joint to achieve the limb flexion. The elbow extension, in contrast, is obtained by lengthening the SAs with an increase in fluid pressure.

**Figure 2:**
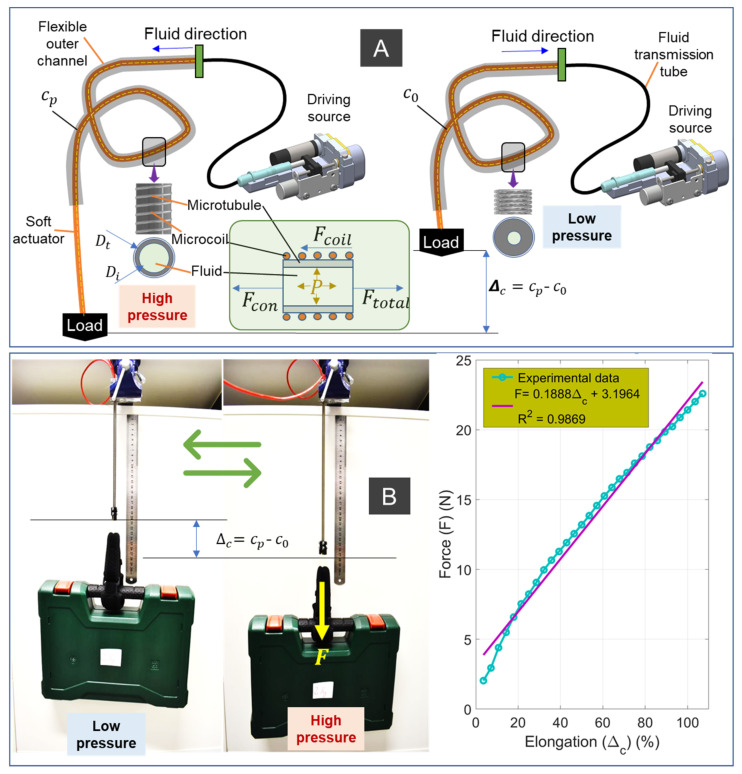
The soft actuator (SA). (**A**) Its working principle when it slides inside a hollow channel and pressure *P* distribution. (**B**) Lifting performance of SA1 (see Table 1, weight of 50 g, outer diameter of 6.35 mm, length of 200 mm), without a flexible outer channel against a load of 2 kg (left panel) and the relation between the elongation and generated contraction force (right panel).

In contrast to conventional fiber-reinforced SAMs, developed SA is a type of long artificial muscle (length is up to few meters, diameter ranges from 0.8 mm to 7 mm) that can be fabricated by the insertion method. This avoids non-uniform distribution of the fiber along its inner silicone tube. Although we previously introduced the fabrication process of this type of SA [33,55], there are several different steps for creating the SA in this work (Figure 2). Firstly, we assembled the guided tube between the hydraulic pressure source (syringe) and the inner rubber tube of the SA by inserting a blunt metal needle into one end of the guided tube (which will be connected to the hydraulic syringe) and a short metal tube into its other end (which will be directly inserted into the hollow channel of the inner rubber tube). To stabilize the connections under high hydraulic pressure, we fastened both sides with super glue and fishing line. Secondly, we inserted the rubber tube inside the channel of the constrained helical coil with excess length on both sides. We then inserted the guide tube with short metal tube to one end of the SA, followed by a tight fastening using super glue and fishing line to form a strong connection. Thirdly, we filled the guide tube and muscle with distilled water to ensure that there are no air bubbles present in the fluid channel. The free end of the SA was then closed by making a tight knot with the excess rubber tube before being tightly fastened to the external helical coil using super glue and fishing line. Finally, rigid 3D-printed parts were used to lock the junctions at both sides of the muscle to increase the device’s durability against high pressure.

Although the SA can transmit force and motion from a distance which is similar to that of conventional flexible cable mechanisms [56,57], it avoids high energy loss between the driving source and the soft actuator due to the use of hydraulic liquid as the transmission element. The SA generates motion via the local extension of individual actuation segment, where the outer helical coil restrains the radial expansion of the inner latex rubber tube, resulting in exclusively axial elongation of the SA under applied hydraulic pressure. The SA generates a contraction force $F_{con}$, by reversing this motion if there is a reduction in hydraulic pressure. Specifically, it stores elastic energy (EE) by extending to a certain length under a high pressure, and it then releases this EE to pull load when this pressure is reduced (Figure 2). Depending on the specific application, the generated force highly relies on the choice of SA materials, its diameter, and the elongated length. Mathematically, the inner rubber tube will be lengthened from initial length $c=c_{0}$ to a length $c_{p}$ with a displacement $\Delta_{c}=c_{p}-c_{0}$, when a hydraulic pressure *P* is supplied to its channel. During the elongation, it accumulates EE, and when the pressure *P* is reduced, it discharges this EE to generate a contraction force $F_{con}$. The higher the applied pressure *P*, the greater the elongation of Δc, thus generating a higher contraction force $F_{total}=F_{con}+F_{coil}-F_{p}$ against a load. It is noted that $F_{coil}=k\Delta_{c}$ is the contraction force of the outer coil, where *k* is the coil stiffness coefficient. $F_{con}=\frac{EA\Delta_{c}}{\left(\Delta_{c}+c_{0}\right)}$ is the elastic force of the inner tube, and $F_{p}=0.25\pi D_{i}^{2}P$ is the driving force generated by fluid pressure *P*, where *E* is the Young’s modulus of the inner silicone tube, $A=0.25\pi \left(D_{t}^{2}-D_{i}^{2}\right)$ is the cross-sectional area of the inner silicone tube once it is inserted into the helical coil, $D_{t}$ is the inner diameter of helical coil, $D_{i}$ is the inner diameter of the silicone tube. 

It is noted that each SA has an upper elongation limit $\Delta_{c,max}$ corresponding to maximum pressure $P_{max}$ to avoid failure during the operation. To mitigate the initial nonlinear dead-zone of the SA, we use a soft rubber tube with a diameter $D_{ot}$, which is larger than that of the inner helical coil diameter $D_{t}$ (Figure 1). The main reason is that the inner tube normally undergoes an initial radial expansion, until it fits tightly into the inner channel of the helical coil, meaning that an increase in input pressure or volume to the inner rubber tube will result in no change in the axial elongation of the muscle. Overcoming this problem will result in the simpler motion control of SA operation. In addition, an initial pre-tension can be applied to the inner rubber tube at the time that it is inserted inside the helical coil, which will significantly increase the contraction force, and reduce the nonlinear hysteresis in a trade-off of high fluid pressure and lower elongation. In order to use the above analytical model, several assumptions are needed. These include: (i) a small inertial force of the fluid; (ii) uniform distribution of the pressure within the fluid channel; (iii) no radial expansion of the inner tube or relative sliding motion between the helical coil and the inner rubber tube; (iv) a Poisson’s ratio for the inner rubber tube of 0.5. This means that the inner rubber tube has a constant material volume during the operation. It is also obvious that the maximum output force generated by the SA can be determined by the stiffness *k* of the outer coil, the muscle elongation $\Delta_{c},$ the material Young’s modulus *E* (which highly depends on the initial pretension applied to the inner tube when it is inserted into the helical coil), and the size of the inner silicone tube A. In addition, the developed SAs have one additional component of generated force ($F_{coil}$) from the outer helical coil, which is an advantage compared to other artificial muscles with similar structures.

### 2.2. Soft Actuator Characterization

**Figure 3:**
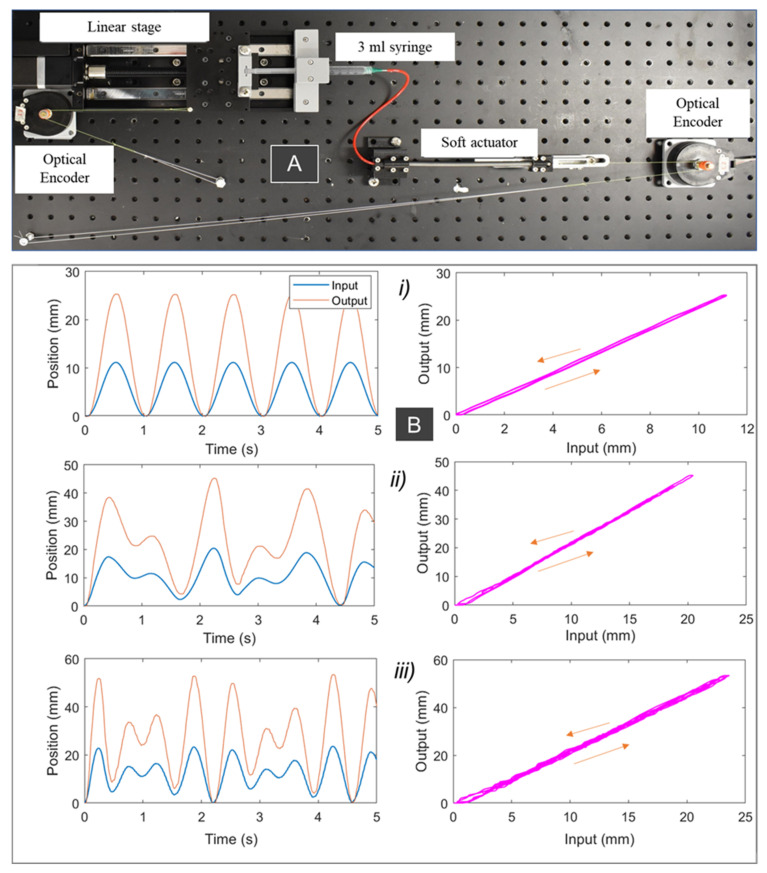
Experimental results for the soft actuator (SA1). (**A**) Experimental setup. (**B**) Different input signals applied to the syringe plunger. They include 1 Hz sine wave input (**i**); two pairings of sine waves (combined frequency of 1 Hz and 1.7 Hz) (**ii**); and two pairings of sine waves (combined frequencies of 2 Hz and 2.7 Hz (**iii**)) with amplitudes scaled to produce 0 to 30% elongation. (**Left panel**) time history of the input and output displacement. (**Right panel**) input displacement versus output displacement (or the SA1 elongation).

A dedicated experimental setup (Figure 3A) was established to investigate the nonlinear hysteresis in the SAs. We applied different input signals to the system to characterize the relationships between the syringe plunger displacement and the SA elongation. The experimental setup consisted of an automated single axis linear stage (Zaber Technologies Inc., Vancouver, BC, Canada) and two optical encoders (US DIGITAL, Vancouver, WA, USA) (Figure 3A). The working fluid (water) was provided via a BD Luer LokTM (3 mL syringe is used for the SA1, and a 0.5 mL syringe is used for the SA2; see Table 1 for detailed specification of the SAs). The barrel of the syringes was clamped on the top of the actuator, while the syringe plunger is positioned on a 3D-printed holder connected to the ball screw mechanism (MISUMI, Tokyo, Japan), to provide the input displacement. A first optical encoder (Omron, Chicago, IL, USA) was employed to record the displacement of the syringe plunger. A fluid transmission tube (Cole-Parmer, Sydney, Australia) was used to connect the syringe and the SA. One end of the SA was fixed onto a 3D-printed base, and the other end sat on a low-friction linear guide (MISUMI, Tokyo, Japan) that ensured longitudinal displacement without any twisting of the muscle. Output displacement of the actuator was recorded by a second optical encoder, while output force was recorded by a force sensor (Mark 10 Series 5, Copiague, NY, USA). The data were decoded and processed using a data acquisition device (QPIDe Data Acquisition Device, Markham, Canada) and MATLAB Simulink (Mathworks Inc., Natick, MA, USA). As discussed in the previous section, the performance of the SA, in terms of hysteresis and generated force, depends on the dimension selection of the inner silicone tube and the helical coil, their materials, and input pressure threshold. Therefore, we fabricated two different prototypes for the SAs (see Table 1). It is noted that the length of the soft actuator does not significantly affect its hysteresis profile and generated force, due to the uniform extension of the SA elements along its axial direction. To reduce the fabrication time, we used different lengths for the two prototypes, where SA1 (Table 1) will be used for both experimental validation and the soft robotic sleeve (see next sections).

**Figure 4:**
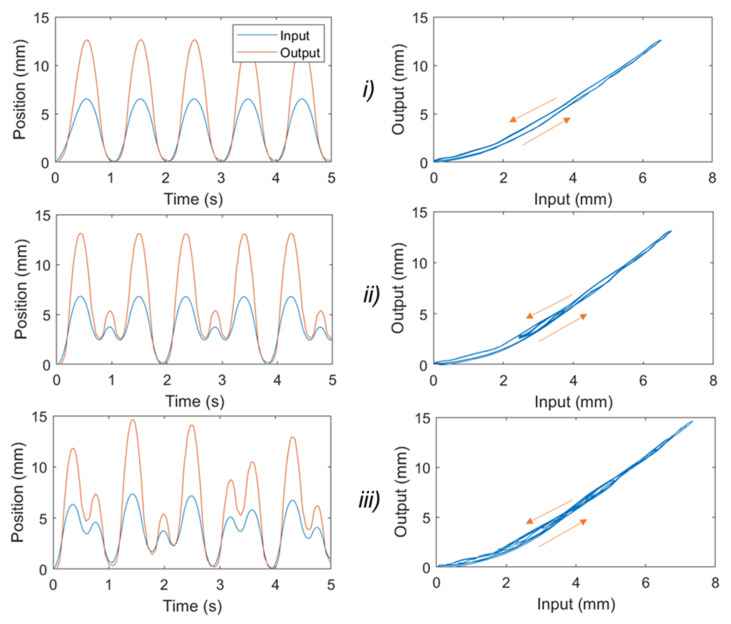
Experimental results for the soft actuator (SA2). Different input signals applied to the syringe plunger. They include 1 Hz sine wave input (**i**); two pairings of sine waves (combined frequency of 1 Hz and 1.5 Hz, (**ii**)); and two pairings of sine waves (combined frequencies of 1 Hz and 1.73 Hz, (**iii**)). (**Left panel**) Time history of the input and output displacement. (**Right panel**) input displacement of the syringe plunger versus the SA2 elongation.

It has been reported that the typical frequency that can provide useful assistance to the upper limbs for most wearable devices is less than 2 Hz [58]. Therefore, we applied different input signals to the syringe plunger via the linear DC motor, which include a 1 Hz sine wave input (Figure 3(Bi)), two pairings of sine waves (combined frequencies of 1 Hz and 1.7 Hz, Figure 3(Bii)), and two pairings of sine waves (combined frequencies of 2 Hz and 2.7 Hz, Figure 3(Biii)). The amplitude of each signal was scaled to produce 0 to 30% of the SA elongation. Experimental results from Figure 3B show that input syringe displacement has an approximate linear relationship with the SA1 elongation. It can be explained by using a strong helical coil, which has a high elastic energy to release once the pressure is reduced. In addition, the inner silicone tube was pre-stretched during the fabrication process, and this contributes to increasing the elastic energy while reducing the nonlinear elastic deformation of the tube. Therefore, the nonlinear hysteresis of SA1 is minimal. However, this reduction has a trade-off in that high hydraulic pressure and applied force are needed to drive the syringe plunger. We also conducted experiments for SA2. Figure 4 shows the time history of the applied input displacement from the syringe plunger (0.5 mL syringe) and the output displacement of SA2. We applied different input signals to the syringe plunger via the linear DC motor, which include 1 Hz sine wave input (Figure 4i); two pairings of sine waves (combined frequency of 1 Hz and 1.5 Hz, Figure 4ii); and two pairings of sine waves (combined frequencies of 1 Hz and 1.73 Hz, Figure 4iii). During the fabrication process for SA2, we avoided applying high pre-tension to the inner tube. The hysteresis characteristic results from Figure 4 revealed that SA2 exhibits a nonlinear hysteresis profile compared to that of SA1 (Figure 3B). This is due to the use of a smaller size for SA2, where the stiffness of the outer coil is smaller compared to that of SA1. In addition, the high deformation of the inner rubber tube under the applied hydraulic pressure resulted in a highly nonlinear hysteresis profile for SA2. However, this type of SA requires a lower pressure to operate, and thus a smaller force to drive the syringe plunger. In practice, one could increase the number of participating SA2s to meet a specific force requirement.

To illustrate the capability of the SAs, we also perform a lifting test whilst recording force measurement with respect to actuator elongation. For the muscle elongation versus tensile force (Figure 2B) experiment, we rigidly fixed one end of the SA1, while its other end is connected to a linear guide via a force gauge (Mark 10 Series 5, Copiague, NY, USA). Before the experiment, we elongated the SA1 to reach around 35% of its initial length. During the experiment, we varied the applied pressure from the syringe to induce contracted force from the SA. The left panel of Figure 2B shows SA1 (weighing 50 g) carrying a 2 kg load. When the muscle was depressurized, it had an initial length *c = c*_0_. When the muscle was pressurized, it expanded axially to $c_{p}=\Delta_{c}+c_{0}$, with no radial expansion, due to the constraint of the outer helical coil. The motion was produced while elastic energy was stored in both the stretched inner tube and the coil. When SA1 was depressurized from the pressurized state, it shortened towards its free length (e.g., from $c_{p}$ to *c*) using the elastic stored energy to pull against the external load. The right panel of Figure 2B shows that there is a linear relation between the SA elongation and output force.

### 2.3. New, Nonlinear Hysteresis Model for a Soft Actuator

Although SA1 exhibits a low hysteresis profile or an approximately linear relation between the input displacement of the syringe plunger and the actuator elongation, a high-pressure source (~3 MPa) is needed to drive the system, and thus a strong linear system is required to generate a high linear force to drive the 3 mL syringe. For some application where the requirement of light weight driving source for wearable purposes is highly desirable, the use of smaller SAs is highly recommended. To fulfil the art for the real-time implementation of the SA at any desired length and scale, we here develop a new nonlinear hysteresis model that can capture the hysteresis profile of the smaller SAs, such as SA2. The new hysteresis model can be used for future compensation control purpose of SA, such as a model-based feedforward control scheme, or nonlinear adaptive control in the case where real-time position feedback is available. The experimental results from Figure 4 show that the nonlinearities between the input displacement of the syringe plunger and the output motion (the elongation of the SA) follow an asymmetric hysteresis profile.

In this work, we introduce a simple yet effective asymmetric hysteresis model to precisely capture the hysteresis loop of the SA2. Although other discrete models such as Preisach and Prandtl–Inshlinskii [51] can be potentially used to capture the hysteresis profile for nonlinear systems, such as the tendon sheath mechanisms [20,21,22,23], their accuracy mainly relies on the use of a high number of hysteresis elements, which requires a large number of model parameters to be identified. Other continuous models, such as the symmetric Bouc–Wen [59,60,61], are not suitable for the SAs, as it can only capture the symmetric profile of the hysteresis where at least seven model parameters must be identified. Like the symmetric Bouc–Wen model [59,60,61], the generalized asymmetric Bouc–Wen model [62] can capture the asymmetric hysteresis profile of nonlinear systems well. However, it still requires ten model parameters in its structure, and this results in more computational time and a complicated control process if a feedforward control algorithm is used. Although we recently introduced an asymmetric hysteresis model that can capture the hysteresis loop of miniature hydraulic muscle [24], this model requires seven model parameters in its structure. To overcome these challenges, we here develop a new asymmetric hysteresis model in the form of differential equations that require only five model parameters in its structure, while precisely capturing the hysteresis profile of SA2. This new asymmetric hysteresis model is expressed by:

(1) $$\left\{\begin{array}{c} \Theta \left(p_{s},\dot{p_{s}}\right)=p_{s}(\beta_{1}+\beta_{2}\tanh(10\dot{p_{s})})\\ \Psi \left(\dot{p_{s}}\right)=\beta_{3}+\beta_{4}\tanh(10\dot{p_{s})} \end{array}\right.$$

(2) $$\dot{\Delta_{c}}=\dot{p_{s}}\left(\Psi \left(\dot{p_{s}}\right)-\delta \;\left(\Delta_{c}+\Theta \left(p_{s},\dot{p_{s}}\right)\right)\mathrm{sgn}\left(\dot{p_{s}}\right)\right)\;=\dot{p_{s}}\left(\beta_{3}+\beta_{4}\tanh(10\dot{p_{s})}-\delta \;\left(\Delta_{c}+p_{s}(\beta_{1}+\beta_{2}\tanh(10\dot{p_{s})}\right)\mathrm{sgn}\left(\dot{p_{s}}\right)\right)$$

where $\beta_{i}\;\left(i=1\;\mathrm{to}\;4\right),\;\delta$ are model parameters that control the shape and size of the hysteresis loop; $p_{s}$ is the input displacement of the syringe plunger; $\Delta_{c}$ is the elongation of SA; the dot at the top of each variable represents the first derivative with respect to time. The hyperbolic tangent which is used to adjust the smoothness of the hysteresis loop is defined by $\tanh(10\dot{p_{s})}=\frac{e^{10\dot{p_{s}}}-e^{-10\dot{p_{s}}}}{e^{10\dot{p_{s}}}+e^{-10\dot{p_{s}}}}$, while the signum function is defined by $\mathrm{sgn}\left(\dot{p_{s}}\right)=\left\{\begin{array}{c} 1\;\mathrm{if}\;\dot{p_{s}}>0\\ 0\;\mathrm{if}\;\dot{p_{s}}=0\\ -1\;\mathrm{if}\;\dot{p_{s}}<0 \end{array}\right.$.

To capture the asymmetric hysteresis profile of the SA, a set of five model parameters $\beta_{i}\;\left(i=1\;\mathrm{to}\;4\right)\;\mathrm{and}\;\delta$ given by Equations (1) and (2) is identified. These parameters are optimized by minimizing the mean square error (MSE) between the proposed model and experimental data via a genetic algorithm (GA). This results in $\beta_{1}=1.2936,\;\beta_{2}=1.8577,\;\beta_{3}=-62.1064,\;\beta_{4}=31.4916,\;\delta =29.9878$.

**Figure 5:**
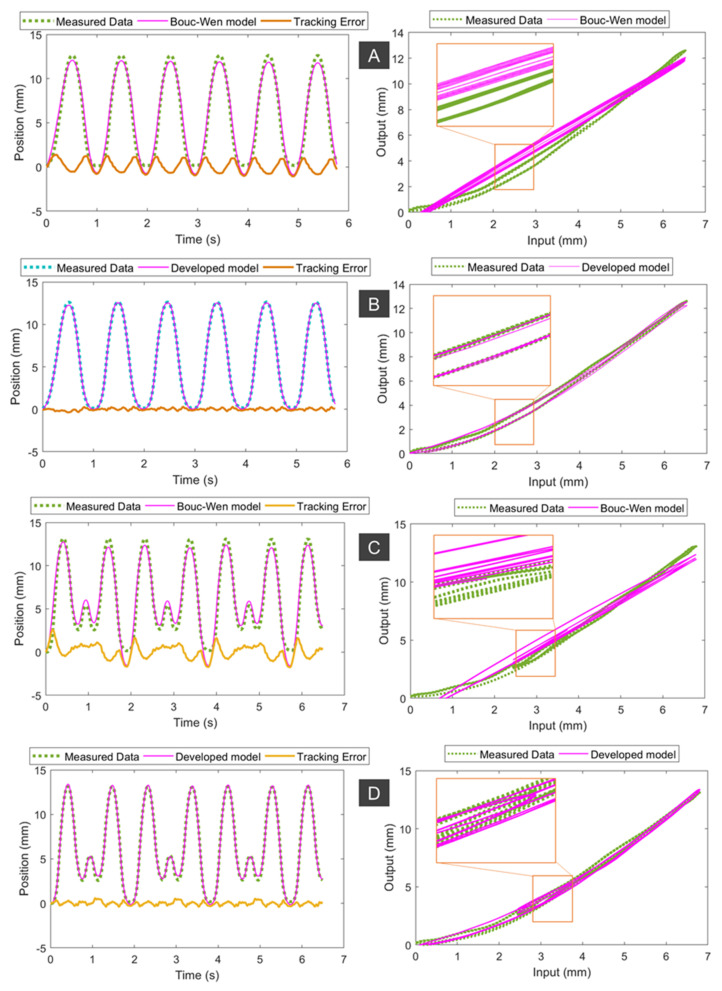
Comparison between the hysteresis models and experimental data using 1 Hz sine wave input signal (**A**,**B**) and combined frequency of 1 Hz and 1.5 Hz (**C**,**D**). (**A**,**C**) The symmetric Bouc–Wen model. (**B**,**D**) The developed asymmetric hysteresis model given by Equations (1) and (2).

The predicted hysteresis curves are given in Figure 5. As shown in this figure, the hysteresis profiles (for single frequency and dual frequencies, Figure 4) are asymmetric in forward and backward direction. The MSEs for the developed model given by Equations (1) and (2) and the symmetric Bouc–Wen model given by [59,60,61] for a single sine wave signal of 1 Hz (Figure 5A,B) are 0.0164 and 0.4557, respectively. Unlike the symmetric Bouc–Wen model, the developed model requires only five model parameters and could much more accurately fit the asymmetric hysteresis profile of SA2. To demonstrate the capability of the developed model, we also performed non-periodic motions (combined sine waves of 1 Hz and 1.5 Hz). Results from Figure 5C,D show that our developed model has a smaller value of MSE = 0.0457 compared to MSE = 0.6252 of the symmetric Bouc–Wen model. These results confirm that our developed model given by Equations (1) and (2) captures the hysteresis profile of the SA2 under various inputs well.

### 2.4. Fabrication of SA-Based Robotic Sleeve with Its Elbow Kinematics and Kinetics

**Figure 6:**
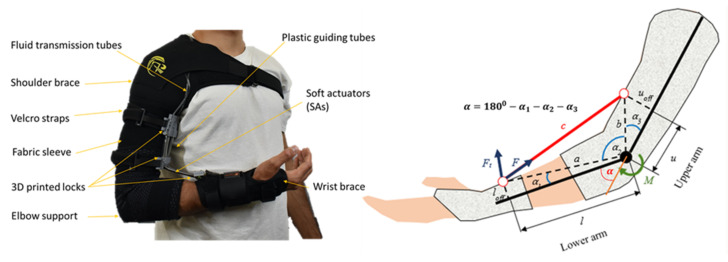
The soft robotic fabric sleeve attached to the elbow (**left**) and its kinematics (**right**).

We fabricated a prototype of a soft fabric robotic sleeve to augment upper limb performance (left panel of Figure 6). As an illustration, we used SA1 (see Table 1) as the main actuation source to drive the soft robotic sleeve. First, we attached the soft fabric sleeve into commercial fabric shoulder and the elbow brace (Amazon, Sydney, Australia) using a sewing machine (FS155, Brother Bridgewater, NJ, USA) to form a contiguous garment. 

The fabric shoulder supported fully encircles the user’s chest to resist the slipping of the fabric frame when tensioned. For a similar purpose, the distal component was routed around the thumb and first finger of the wearer. This was created by removing the aluminum support from a commercial wrist orthosis (Amazon, Sydney, Australia). Moreover, 3D-printed locks were used to secure the SA1s to the fabric sleeve. The upper and middle locks were attached to the arm using Velcro straps (Bunnings, Perth, Australia), while the lower lock was sewn into the non-stretchable fabric of the distal component to prevent unpredictable and undesirable deformation caused by the tensile forces applied by the muscles. To avoid the misalignment of the 3D-printed locks and unwanted forces or moments acting on the arm, a fabric pattern was embedded into the elbow sleeve to indicate the correct positioning of the locks. This design enabled a quick and simple replacement of the SA by opening the locks. A soft liquid metal-based strain fabric sensor (see next section) was attached along and onto the surface of the soft actuators, with its ends attached to the Velcro straps.

We also developed a simple kinematic and kinetic model, with illustrated parameters for the elbow (see the right panel of Figure 6). Briefly, when the elbow joint was fully extended, the elbow angle α is assumed to be zero, where $\alpha =180^{0}-\alpha_{1}-\alpha_{2}-\alpha_{3}$ and $\alpha_{2}$ changed in accordance with the elbow joint, while angles $\alpha_{1}$ and $\alpha_{3}$ stayed unchanged for the predetermined values of the lower and upper arm lengths (*l* and *u*) and the corresponding offset from the underlying bone ($l_{off}$ and $u_{off}$). The elbow kinematic and kinetic model can be expressed by:

(3) $$\left\{\begin{array}{c} \alpha_{1}=\tan^{-1}\left(\frac{l_{off}\;}{l}\right)\\ \alpha_{3}=\tan^{-1}\left(\frac{u_{off}\;}{u}\right) \end{array}\right.$$

(4) $$a=\sqrt{l_{off}^{2}+l^{2},\;}b=\sqrt{u_{off}^{2}+u^{2},}$$

(5) $$\left\{\begin{array}{c} c^{2}=a^{2}+b^{2}-2ab\cos\alpha_{2}\\ \alpha_{2}=\cos^{-1}\left(\frac{a^{2}+b^{2}-c^{2}}{2ab}\right)\; \end{array}\right.$$

(6) $$M=aF_{t}=ab\frac{F}{c}\sin\alpha_{2}$$

Using the measured analogue signal from the soft fabric sensor (see next section), we can obtain the SA length *c* from which $\alpha =180^{0}-\alpha_{1}-\alpha_{2}-\alpha_{3}$ can be computed using the cosine law given by Equations (3)–(5). The torque *M* contributed by the soft upper limb support can be calculated from the perpendicular component of the muscle tension $F=nF_{total}$ with respect to segment *a* (forearm) (see Equation (6)), where *n* is the number of SA1 units used in the robotic sleeve, and $F_{total}$ is the generated force of each SA1. It is noted that this model can be used in the future precision control of the wearable fabric sleeve.

### 2.5. Liquid Metal-Based Soft Fabric Sensor for Bending Motion Detection

**Figure 7:**
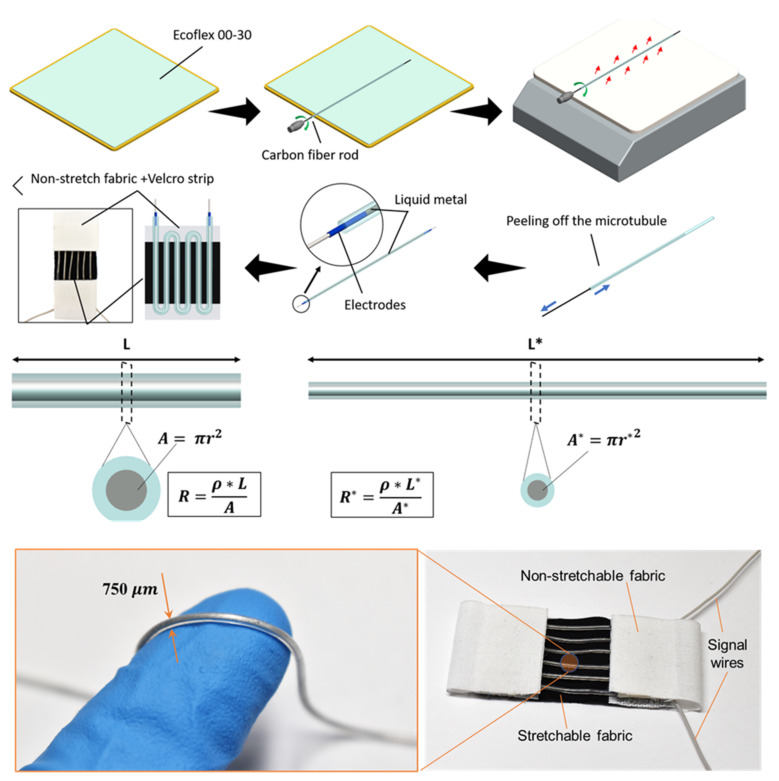
The fabrication process and prototype of the liquid metal-based soft fabric sensor.

To detect the bending motion of the elbow joint, a soft liquid metal-based fabric strain sensor was developed (Figure 7). The new sensor was designed in a way that it could be directly integrated into the soft robotic sleeve. The new sensor was comprised of elastic fabric substrates and liquid metal-filled soft microtubules. The microtubules were fabricated by a roll-coating method with liquid silicone elastomers. Briefly, the platinum-cured soft elastomer (Smooth-On, Inc., Macungie, PA, USA) was mixed with a weight ratio of 1:1 (part A: part B) and laminated onto a metal plate by a thin-film applicator (Zehntner GmbH Testing Instruments, Sissach, Switzerland) to approximately 750 μm in thickness. A carbon fiber rod (Composite Store Inc., Tehachapi, CA, USA) was then rolled on the uncured silicone surface using a hand drill, and subsequently heated over a hot plate. The silicone quickly cured under the high temperature, forming thin-walled microtubules that can be safely peeled off the carbon fiber rod. Different rod diameters could be used to control the inner diameter of the microtubules. The wall thickness and therefore the outer diameter of the microtubules were also adjusted by varying the number of rolling layers. In this work, the microtubules were fabricated with an ID of 450 μm, an OD of 750 μm, and a length of 250 mm. Once the soft microtubule was created, it was then filled with EGaIn liquid metal (Sigma Aldrich, Sydney, Australia) by a miniature syringe (BD MEDICAL SYRINGE, Franklin Lakes, NJ, USA) and a 25 G blunt needle (Element14, Sydney, Australia), and sealed at both ends by electrodes made from 40 AWG wires (Element14, Sydney, Australia) using superglue (Super Glue, Ontario, CA, USA). The EGaIn-filled microtubule was then arranged into a serpentine shape with 3 loops on a stretchable fabric (black color in Figure 7) and secured at both ends with Velcro strips and silicone glue. Two more pieces of non-stretchable fabrics (white color in Figure 7) were sewn to both ends of the stretchable fabric substrate, enabling the middle section to be the only part that experiences strain during the movement of the arm. Figure 7 describes, in detail, the design of the fabric strain sensor.

The fabric strain sensor works as a piezoresistive sensor, where its length change corresponds to the change of its resistance defined by $R=\rho \frac{L}{A}$, where the liquid metal resistivity $\rho =2.9\times 10^{-6}\;\Omega \mathrm{cm}$, *A* is its cross-sectional area, and *L* is its length. Upon the elongation of the muscles, the fabric strain sensor also increases in its length, from *L* to L*, and decreases in its cross-sectional area, from $A=\pi r^{2}$ to $A^{*}=\pi {\left(r^{*}\right)}^{2}$, where $r,\;r^{*}$ are the radius of the liquid metal channel of the sensor. This deformation results in a change in the sensor resistance, from *R* to $R^{*}=\rho \frac{L^{*}}{A^{*}}$, as depicted in Figure 7.

### 2.6. Characterisation of Soft Liquid Metal-Based Fabric Sensor

**Figure 8:**
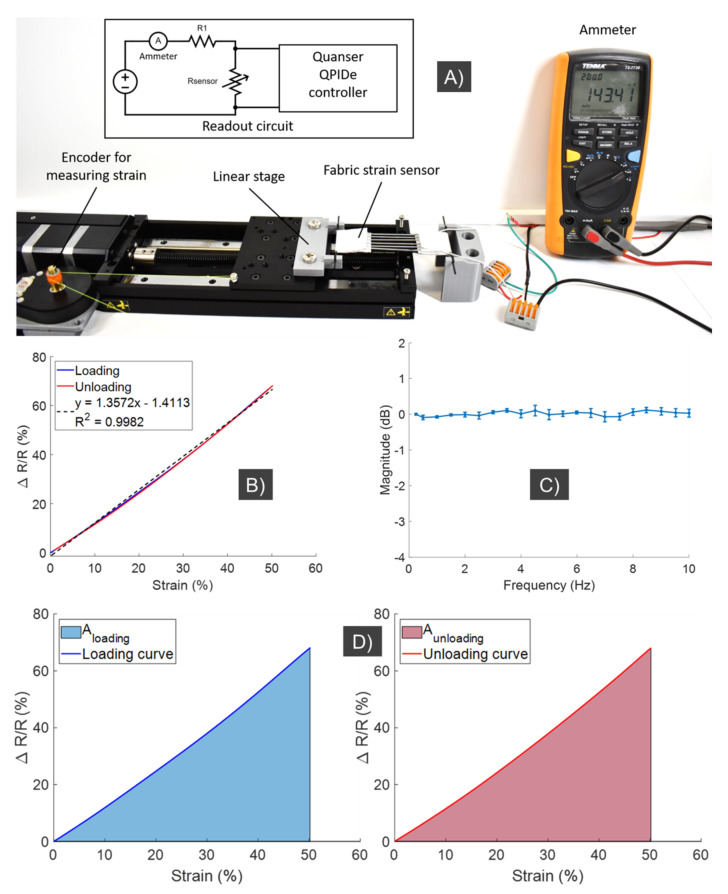
Characterization of the soft fabric sensor. (**A**) Experimental setup for characterization of the fabric sensor. (**B**) Change in resistance versus strain. (**C**) Frequency response. (**D**) Degree of hysteresis (DH) calculation.

The fabric strain sensor was first characterized in a separate experiment, with a setup depicted in Figure 8A. The inset image of Figure 8A describes the schematic of the readout circuit that was used to acquire sensor readings. Using this experimental setup, the fabric strain sensor was subjected to elongation (loading) and retraction (unloading) cycles at 0.2 Hz by an automated linear stage (Zaber Technologies Inc., Vancouver, BC, Canada). The relationship between normalized changes in the resistance, ΔR/R, of the sensor and its strain was collected and displayed in Figure 8B. In order to characterize the hysteresis in the sensor response, the degree of hysteresis (DH) is quantified by the following equation:

(7) $$DH=\frac{A_{loading}-A_{unloading}}{A_{loading}}100\%$$

where $A_{loading}\;$ and $A_{unloading}$ are the areas of resistance loading and unloading curves, respectively, or the areas under the loading and unloading curves of the resistance change-strain relationship (see Figure 8D).

According to Equation (7), the lower DH value indicates less hysteresis in the sensor response, and therefore, more precise strain-resistance relationship. The DH value of the fabric strain sensor fabricated in this paper was calculated as 0.8%, which indicates a low hysteresis response. In addition, the strain-resistance relationship of the fabric sensor can also be seen to be highly linear, demonstrating a linear regression with R^2^ = 0.9982. This result indicates that a simple calibration and readout method is possible to obtain the strain values, and subsequently bending angles, directly from the sensor’s resistance changes. It is noted that the bending angle α is estimated based on the strain *c* (or elongation) of the muscle. Detailed calculation is shown in Equation (3) to Equation (6) and Figure 6. The strain or elongation *c* is directly estimated based on the resistance change (see Figure 8B, where the relation between the resistance change and strain is almost linear). The fabric strain sensor was also put through a frequency response experiment, with loading stimuli of at least 10 Hz and three repetitions for each frequency. It is noted that the sensor could operate at higher frequencies. However, the soft wearable device used here operated at frequencies which were less than 5 Hz, and therefore we only tested within this range up to 10 Hz. The results demonstrated in Figure 8C show that the sensor can work well with stimuli of at least 10 Hz, with minimal attenuation in the signal readings, and therefore it is compatible with the support robotic sleeve.

### 2.7. Characterization of the Soft Robotic Fabric Sleeve for Upper Limb Augmentation

We also carried out experiments to validate the soft robotic sleeve to augment upper limb performance. This experiment aimed at demonstrating that the developed system is stable and could provide necessary assistance to the upper limb. Subjects were provided all written informed consents prior to the experiment, and the procedures were approved by the Institutional Review Board, University of New South Wales. It is noted that this device can be worn as a conventional sleeve, where the shoulder and elbow supports are formed into a continuous garment with the soft fabric sleeve using a sewing machine. To prevent the fabric frame from slipping under high tension, the shoulder support fully covered the wearer’s chest, while the wrist support also wrapped around the user’s wrist, thumb and first finger. We used electromyogram (EMG) electrodes (ADInstruments, Dunedin, New Zealand), which were attached below the soft upper limb sleeve to monitor the EMG signals. Two conditions were applied during the experiments: (i) the wearers without holding any load started with a pose of 90° arm flexion. The wearers then extended to 0° and flexed back to 90°; (ii) the wearer carried a 1.8 kg weight with a pose of 90° arm flexion. Then, the wearer extended to 0° and flexed back 90°. Each moving cycle was repeated 6 times. Three conditions were tested: (1) the users wore the soft robotic sleeve without actuating the SA through the whole movement (denoted as SA w/out act); (2) the wearer received support from the soft robotic sleeve with the SA pressurized during the 90° extension and depressurized during the 0° to 90° flexion (denoted as SA w/act); and (3) the wearers did not wear the soft robotic sleeve (denoted as no SA). The EMG electrodes were placed in identical positions for all three configurations. 

**Figure 9:**
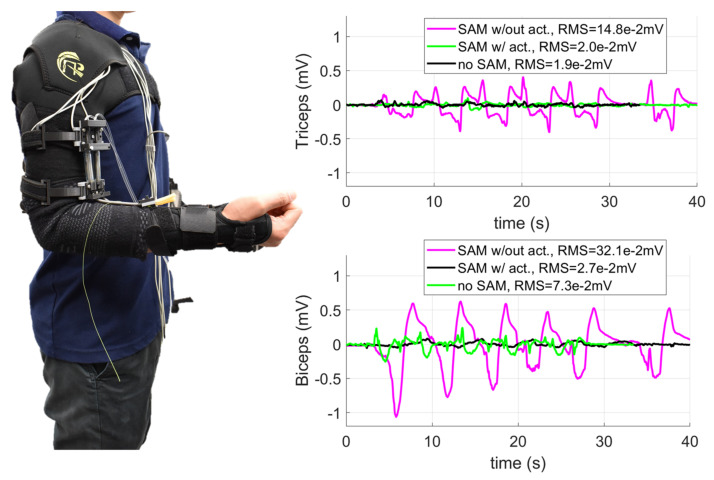
Validation of the soft robotic fabric sleeve for upper limb augmentation of a subject with EMG measurements and no load. (**Left panel**) Experimental setup for EMG measurements with no load. (**Right panel**) EMG measurements.

**Figure 10:**
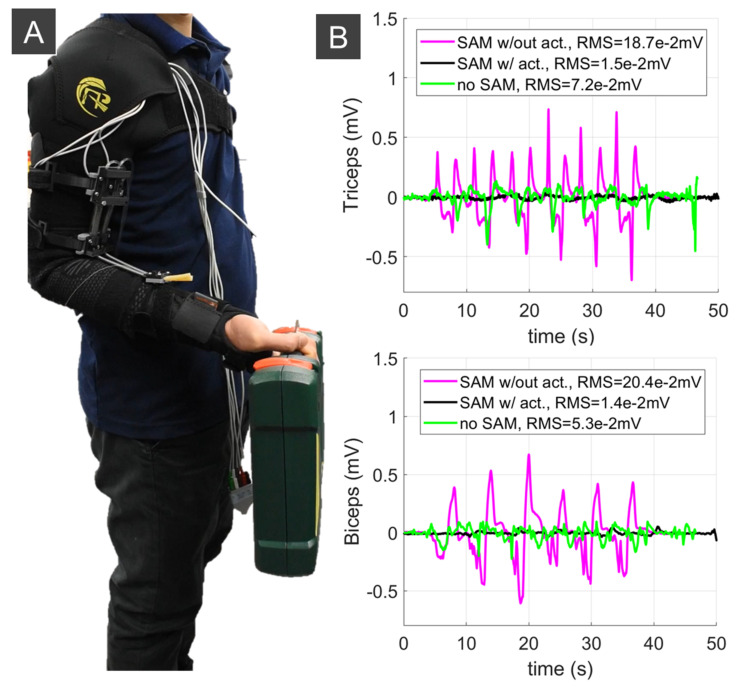
Validation of the soft robotic fabric sleeve for upper limb augmentation with EMG measurements and load of 1.8 kg. (**A**) Experimental setup for EMG measurements. (**B**) EMG measurements.

The results in Figure 9 and Figure 10 indicate that the soft robotic sleeve reduced the workload of the subject’s biceps and triceps compared to the configurations with no SA, and without actuating the SAs. For example, without lifting any load, the root mean square error (RMS) for the triceps and biceps was reduced around 7 times (from RMS = 0.148 mV to RMS = 0.02 mV) and 12 times (from RMS = 0.321 mV to RMS = 0.027 mV) if the soft robotic sleeve is activated, respectively. For experiments when the wearer held a load, there was a significant reduction in the RMS (around 12.5 times for the triceps from RMS = 0.187 mV to RMS = 0.02 mV and 14.5 times for the biceps from RMS = 0.204 mV to RMS = 0.014 mV) once the soft robotic sleeve was activated. It is noted that the first configuration (i.e., SA w/out act) had significantly higher muscle activation, because the wearer’s arm not only had to carry the 1.8 kg weight, but also resist the non-actuated SAs.

## 3. Discussion

According to [1,2], the actuators used in exoskeletons should be lightweight, capable of producing precise motion, have high operating bandwidth, and be able to deliver large torques. Our developed SAs are soft, lightweight, and highly compliant. They were tested at frequencies ranging from 1 Hz to 2 Hz to prove their capability of operating at the frequency bandwidth used in most assistive elbow exosuits [18,19,30,63]. The bending independent behavior represents a big advantage over the widely adopted Bowden cable transmission, whose configuration-dependent hysteresis is found to degrade control performance and limit its application [58,64]. Our developed muscles, in contrast, are not affected by the change of its configuration from the driving source to actuation source or a constant hysteresis profile, regardless of its transmission paths (see [24] for more details). The new SAs are flexible and lightweight, where an SA weighing 50 g and with an OD = 6.35 mm could lift a load of at least 2 kg. Compared to other artificial muscles, the developed SAs are scalable, have high aspect ratios (length/diameter ~ 314, Figure 2), and could induce a strain up to 245% [33].

The characterization of the SAs showed an approximate linear relation between the elongation and generated contraction force, which introduces a great flexibility to control the generated force via position information. For a large SA (OD > 6 mm), there is an approximate linear relation between the displacement of the input syringe plunger and the SA elongation, in a trade-off of high hydraulic pressure and applied driving force. For the smaller SA (OD~3.18 mm), there exists a nonlinear hysteresis loop between the syringe plunger displacement and SA elongation. However, the smaller SAs require low hydraulic pressure (<1 MPa) to actuate the system, and thus require less power. In some studies, output feedback methods have been used to compensate for hysteresis, but this requires additional sensors that increase device size [21,65]. Since the friction between the hollow tube and the SA does not vary with the bending angle of the SA, as it does in a Bowden cable mechanism, the developed SAs exhibit better transmission efficiency and ease of control when compared to tendon-driven actuators, due to the use of a hydraulic source. Moreover, the flexibility of the SAs allows for complex configuration, such as woven structures that can achieve larger displacements compared to a single muscle, which is challenging with traditional McKibben muscles.

To capture the nonlinear hysteresis loop for the smaller SAs, a novel asymmetric hysteresis model was developed. The new model only requires five model parameters in its structure to closely predict the nonlinear hysteresis loop. Compared to other traditional models, such as the symmetric Bouc–Wen model, which requires a higher number of model parameters, our developed model requires fewer computational times and offers an ease of control if a feedforward compensation-based control scheme is used. The new model also precisely captures the nonlinear hysteresis of the SA for different input signals at different working velocities. Because there is a linear relation between the contraction force and the SA elongation, these results demonstrate that our developed hysteresis model is well suited for force control. This will be the subject of the future works with applications in soft wearable devices such as flexible surgical robotics or smart garments [66,67].

We have also fabricated and successfully validated soft liquid metal-based fabric sensors that can monitor the position (elongation) of the SA. The new fabric sensors can be used as potential candidates to monitor the upper limb and lower limb motion for gait posture detection, or provide real-time motion feedback for advanced control purpose, such as nonlinear adaptive control to deal with nonlinear disturbances and uncertainties from the surrounding environments. To demonstrate the usefulness of the SA, we also created a soft robotic fabric sleeve that can provide useful assistance to the wearer’s upper limb. As an illustration, we use only two SAs in the robotic sleeve structure, and this device could assist the upper limb, without requiring any additional power from the body. Depending on the power augmentation needs, more SAs could be added to increase the support for tasks which require a high load. To drive the SA, a small amount of fluid (~2 mL of water) is required, and this offers a miniaturization for the driving source that can be worn by the users. It is noted that the actuation stage is also scalable, because the fluid syringe functions as a fluid reservoir that can be changed in size according to the available force of the driving source. For example, a smaller syringe would require a smaller applied force to the plunger, which can be generated by a smaller linear stage. Experimental results also revealed that the soft robotic sleeve significantly reduced the workload of the subject’s biceps and triceps compared to the configurations with no SA and without actuating the SAs.

It is noted that, in this paper, we implemented the developed soft muscles for a wearable assistive device for upper limb augmentation, with several advantages compared to our works [33,55]. Firstly, we investigated different outer constrained coil for the soft muscles, where a linear relation between the input and output motion can be achieved if a stiffer coil is used, and vice versa (see Figure 3 and Figure 4). Secondly, we developed a novel nonlinear hysteresis model (See Section 2.3), which could capture the nonlinear hysteresis profile for the soft muscle, with a smaller number of model parameters (five) compared to previous works (seven) [55]. Thirdly, we developed a new soft fabric liquid metal skin sensor that can monitor the elongation or strain *c* of the soft muscle for future feedback control purpose. Finally, while previous works only focused on the use of a single muscle, in this paper, we developed an array of soft muscles to actuate the wearable fabric sleeves that could provide useful assistance to the wearer. In addition, these muscles were combined with the outer sheath to enhance the desired elongation or working range of the bending elbow, which were not reported in previous works.

However, one of the major limitations of the presented soft robotic sleeve is the use of a tethered driving hydraulic source which limits its use for home rehabilitation applications. The target population of such a soft exoskeleton has been identified to be individuals with some physical disability that needs assistance during activities of daily living (ADLs), such as drinking from a mug, combing the hair, eating with a spoon, or answering the phone. Therefore, future development of the current soft robotic sleeve must involve a portable hydraulic source that can be worn by the users, such as the works proposed by [68,69,70]. During the mannequin experiment, the device showed the ability of maintaining the arm posture and a smooth natural elbow flexion motion. The stretch sensor and the kinematic and nonlinear hysteresis model demonstrate the possibility of applying force control algorithms to actuate the soft upper limb in a closed-loop manner.

## 4. Conclusions

This paper describes the design and fabrication process for a new class of soft artificial actuators and soft liquid metal sensors that can be integrated into a soft upper limb suit. The soft muscle actuators are lightweight, have high operating bandwidth, are capable of producing precise motion, and could deliver a large amount of torque (around 23 N per muscle at 30% of its elongation). Similarly, the soft sensor can measure distance/displacement between two points (one at forearm and the other at upper arm) from which the joint kinematics of the soft upper limb suit (elbow joint angle) can be inferred. The new hysteresis model has also been introduced to capture the nonlinearity of smaller SAs that can directly benefit lightweight wearable devices. In addition, this new hysteresis model can benefit the future development of a feedforward compensation-based control law to further promote the applicability of the system. The assistive sleeve shows great potential in assisting people with disabled upper limbs in performing ADLs, or in rehabilitation applications outside of clinical settings, such as in the patients’ home, with benefits including lowering the cost of care and improving accessibility to care. In future work, feedback motion control that recognizes the intention of the user should be developed for assistance application, such as nonlinear adaptive controls. In addition, the soft sensor and linear DC motors should also be re-designed to provided portable and compact wireless signal communication and control for better use in practice. Furthermore, the user studies should be carried out on disabled people to demonstrate the effectiveness of the approach. The validation of the soft robotic sleeve should also be carried out in the presence of disturbances, such as the varying load applied against the wearer’s hand. Finally, the developed concept and prototypes in this work can be extended to support the lower limbs and the whole body towards a complete soft wearable suit to augment human performance.

## Figures and Tables

**Table 1:** Physical properties of the SAs used in experiments.

Prototypes	Inner Rubber Tube	Outer Helical Coil
SA1	Manufacturer: Latex rubber tube (McMaster-Carr Supply Co., Elmhurst, IL, USA) OD: 6.35 mm ID: 3.175 mm Durometer: 40A	Manufacturer: Spring-Tempered Steel (McMaster-Carr Supply Co., Elmhurst, IL, USA) OD: 6.35 mm ID: 5.18 mm Length: 200 mm
SA2	Manufacturer: Latex rubber tube (McMaster-Carr Supply Co., Elmhurst, IL, USA) OD: 3.18 mm ID: 1.59 mm Durometer: 40A	Manufacturer: Spring-Tempered Steel (McMaster-Carr Supply Co., Elmhurst, IL, USA) OD: 3.18 mm ID: 2.51 mm Length: 20 mm

## List of Abbreviations

**Abbreviation**	**Meaning**
ADL	Activities of Daily Living
FFA	Flexible Fluidic Actuator
SMM	Shape Memory Material
EAP	Electroactive Polymer
PAM	Pneumatic Artificial Muscle
PPAM	Pleated PAM
IPAM	Inverse PAM
SMA	Shape Memory Alloy
SMP	Shape Memory Polymer
DEA	Dielectric Elastomer Actuator
IPMC	Ionic Polymer–Metal Composite
SAM	Soft Artificial Muscle
SA	Soft Actuator
LM	Liquid Metal
EGaIn	Eutectic Gallium Indium
EE	Elastic Energy
MSE	Mean Square Error
GA	Genetic Algorithm
EMG	Electromyogram
DH	Degree of Hysteresis
RMS	Root Mean Square
ID	Inner Diameter
OD	Outer Diameter
